# Computational Termochemistry Study of the C_80_ Isomers and Their Endo Lanthanum Complexes by Applying Homodesmotic and Isodesmic Reactions

**DOI:** 10.3390/molecules171214588

**Published:** 2012-12-07

**Authors:** Citlalli Rios, Roberto Salcedo

**Affiliations:** Instituto de Investigaciones en Materiales, Universidad Nacional Autónoma de México, Circuito Exterior s/n, Ciudad Universitaría, 04510, Coyoacán, Ciudad de México, Mexico

**Keywords:** fullerenes, C_80_ isomers, theoretical calculations, endohedral complexes, homodesmotic and isodesmic reactions

## Abstract

C_80_ is a fullerene species which appears in different isomeric configurations. A general homodesmotic reaction previously designed to study the energy of fullerenes was implemented, in order to analyze the energy of this family of isomers. These results concur with some of the experimental data, but energy differences referring to all the configurations yield novel propositions about their particular behavior. The corresponding lanthanum complexes are also analyzed here and a new isodesmic reaction was designed for this particular case.

## 1. Introduction

Large fullerenes other than C_60_ and C_70_ have not been industrially produced because they are difficult to obtain and because possible applications are currently only at the level of research [1]. However C_80_ fullerenes have attracted great interest because they represent almost the smallest cages which can be used for the production of endohedral organometallic compounds [2], although there are cases with smaller fullerenes [3]. 

Following the “isolated pentagon rule” [4,5], there are seven possible configurations for C_80_, which have the following point group symmetry: I_h_, D_3_, D_2_, D_5h_, D_5d_ and two C_2v_. However, many authors refer to them only as a particular nomenclature of letters [6]. Out of all these isomers, only two have been prepared and characterized as pristine structures: these are D_2_ [7] and D_5d_ [8]. Likewise, one report exists referring to another isomer which was detected as a charged species and which is also one of the C_2v_ forms [9]. It is also important to mention that endohedral organometallic complexes from the D_5d_ [10] have also been identified from the D_5h_ [11] and I_h_ [12] isomers (it should be mentioned that these represent the most unstable conformations which can exist as isolated fragments).

The relative energy among all isomers has been calculated using semi-empirical methods [13]. The results including the relative difference (in kcal/mol) when compared to the most stable example (*i.e.*, D_5d_) are as follows: D_5d_ (0), D_2_ (2.3), C_2v_ (23.6), D_3_ (36), C_2v’_ (38.5), D_5h_ (49.9), I_h_ (102).

The main purpose of this work is to present an alternative energy analysis for the isolated isomers, as well as for the endo complexes where two lanthanum ions are found within the cages. The analysis in this work is carried out with reference to the thermochemistry results, taking advantage of a homodesmotic reaction that was specially designed for the pristine fullerenes, together with a new isodesmic reaction specifically designed by applying on the endo complexes of C_80_ species. In fact the results are qualitatively similar to those previously presented by Akasaka and his coworkers [13,14]. However, the quantitative results are different. The differences in the Molecular Orbitals of all conformations are also discussed.

## 2. Results and Discussion

### 2.1. Pristine Fullerene

The problem of the relative energies associated with the isomers of C_80_ has been previously treated by other groups. A very interesting experimental study was carried out by Rojas and his coworkers in which they obtained molar standard enthalpies of formation of several fullerene families and compared these with theoretical results [15], C_80_ was not included in the experimental work, but the theoretical data included some results about it that were used only for comparison with other cages. A very important theoretical work about this topic is the one by Furche and Alhrichs [16]. These authors calculated all the members of the family using functional density methods and even show theoretical nuclear magnetic resonance and electronic absorption spectra. It is important to cite also the ZINDO calculations by Nagase [17] and his coworkers who calculated the excited states for all the isomers showing that these states are very important for the description and comparison of all the family and its derivatives. Aihara and Yoshida [18] proposed an index of kinetic stability based on the HOMO-LUMO energy gap for fullerenes and the family of C_80_ is considered into the whole study. Finally Cioslowski and his coworkers [19] have obtained accurate standard enthalpies of several fullerenes (including the C_80_ isomers) by means isodesmic interconversion reactions from B3LYP/6-31G* results although the work of Slanina and his group [20] about heat of formation should be cited, even considering that C_80_ is not included in the study. 

The problem concerning the stability of these conformations derives from the I_h_ structure. It is likely that I_h_ is the more symmetric species, almost all the lower symmetries are subgroups of the I_h_ point group (with the exception of D_5h_) [21,22,23,24]. Identifying an isomer from the same symmetry point group as the classical C_60_ fullerene suggests that this will represent the most stable conformation; however this is not the case, actually the C_80_ experiments a Jahn-Teller distortion and as a result arise species with lower energy and less symmetry [1,24]. An analysis of the frontier molecular orbitals makes it possible to follow this distortion in all cases. In all the molecular orbital diagrams displayed, the energy values are presented in units of eV. It is also important to indicate that this analysis, as well as the one corresponding to the endohedral complexes only takes into account six isomers. The results obtained for both C_2v_ structures are very similar, so that only one of these was included in the study.

**Figure 1:**
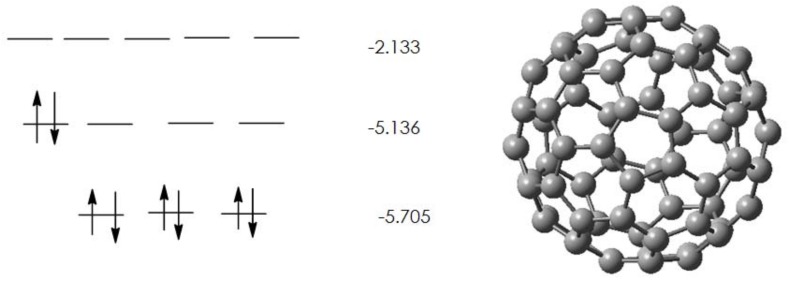
Frontier molecular orbitals and shape of I_h_ isomer (all values in eV).

Figure 1 shows the conformation and the frontier molecular orbitals for the C_80_ I_h_ isomer. This member of the family has the most pronounced symmetry out of the entire set. Curiously the HOMO seems to be a single orbital, however it is very near the LUMO, with such a closeness that it can be considered as a four-folded degenerated set with an occupation of only a pair of electrons, this strange situation was previously found by Manolopoulos and Fowler by means a simple Hückel calculation on the I_h_ C_80_ structure [25], showing both the intrinsic instability of this species and the tendency to go to the Jahn-Teller distortion, a similar scheme was previously discussed by Nagase and coworkers [26]. Therefore the energy difference between HOMO and LUMO is considered from this pseudo degenerated set to the next unoccupied molecular orbital and this is 3.03 eV.

**Figure 2:**
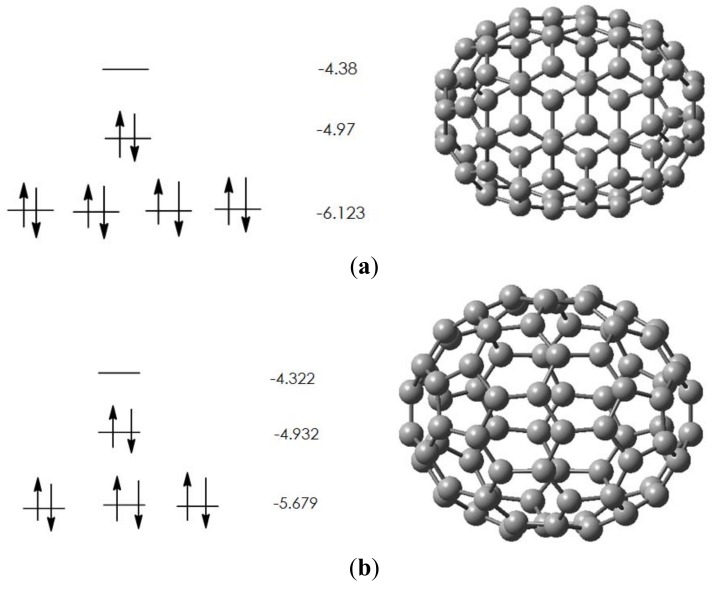
(**a**) Frontier molecular orbitals and shape of D_5d_ isomer (all values in eV). (**b**) Frontier molecular orbitals and shape of D_2_ isomer (all values in eV).

Figure 2 shows the conformations and frontier molecular orbitals of those isomers expected to be more stable, *i.e.*, D_5d_ and D_2_. Both point groups are subgroups of the I_h_ [24]; therefore it is likely that this distortion will give place to new conformations. In the first case, HOMO (−4.97 eV) and LUMO (−4.38 eV) are single orbitals and the energy gap is 0.59 eV. HOMO-1 is a double degenerated set which would appear to belong to the e_u_ irreducible representation, although this set joined to the next two orbitals might be considered to consist of an accidental four degenerated set, because the next three orbitals are so close in terms of their energy (−6.123 eV on average). However the D_5d_ point group has no four-fold degenerated irreducible representations, therefore this is the source of the accidental degeneracy. This arrangement seems to be less crowded than the same one in the I_h_ isomer. The frontier molecular orbitals are very near; this feature suggests it is possible to find a near excited state, a triplet was predicted by a calculation with geometry optimization at the same level and it is above in energy but very near the ground state (2.07 eV), this result is in agreement to the similar study by Furche and Ahlrichs [16].

In the case of the D_2_ isomer, there are no degenerates; HOMO (−4.932 eV), HOMO-1 (−5.679 eV) as well as LUMO (−4.322 eV) are all single orbitals, moderately separated and the energy gap is large (0.61 eV). It is important to note that HOMO-2 represents a set, where once again there is an accidental triple degeneracy (taking into account that the D_2_ point group has no degenerated irreducible representations) and is placed in a position so close to the upper orbitals (1.49 eV), that it is able to interact in an electronic exchange. 

**Figure 3:**
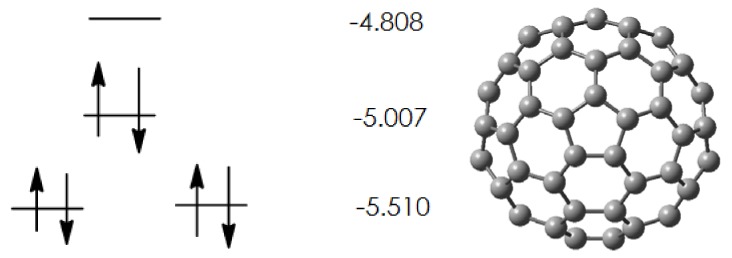
Frontier molecular orbitals and shape of C_2v_ isomer (all values in eV).

The C_2v_ isomer presents an arrangement that can be observed in Figure 3, also displaying the shape of this isomer. This molecule also arises from the I_h_ distortion, the HOMO is a single orbital (−5.007 eV) as is LUMO (−4.808 eV), however the HOMO-1 (−5.51 eV) set is interesting because this point group has no double degenerated irreducible representations; instead this orbital is formed by two eigen functions representing another case of accidental degeneracy and manifesting a very small energy gap (0.199 eV). These isomers are expected to be less stable than their D_5d_ and D_2_ counterparts.

**Figure 4:**
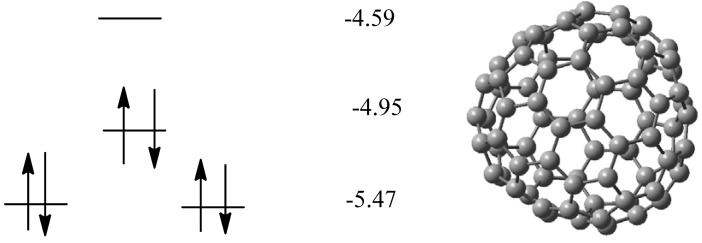
Frontier molecular orbitals and shape of D_3_ isomer.

D_3_ isomer manifests a similar situation to the C_2v_ isomer (see Figure 4). It has the HOMO and LUMO as single orbitals, with energy values of −4.95 eV and −4.59 eV respectively. Once again, there is an accidental degeneration in HOMO-1 (−5.47 eV) and the frontier orbitals are very similar to each other, with an energy gap of 0.36, this so small energy difference between HOMO and LUMO suggest that this species should have a triplet state as in the D5d isomer analyzed above and indeed Furche and Ahlrichs [16] propose that this species should polymerize forming insoluble solids, therefore the triplet state was also calculated within the same method and conditions and the proposition is assessed because the triplet state has a very small energy difference (1.87 eV) with the respect the ground state.

**Figure 5:**
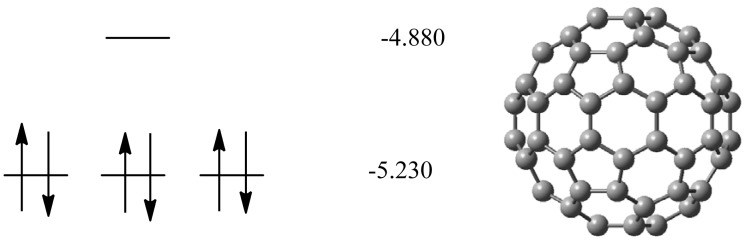
Frontier molecular orbitals and shape of D_5h_ isomer (all values in eV).

The case of the D_5h_ isomer should be treated as a separate topic because this is the other case of large instability as well as the I_h_ case (see Figure 5). Indeed the conformation has such a limited stability that maybe it is possible to suggest a kind of bond-stretch isomerism (π-tautomerism) as that suggested by Fowler [27] giving place to other more stable isomer. The LUMO (−4.88 eV) is a single orbital, similar to the most recently described cases, but now HOMO (−5.23 eV) presents a new case of an accidental degenerated set, comprising a triple folded set that does not correspond to any irreducible representation in the D_5h_ point group. The energy difference is 0.35 eV, suggesting very strong semiconductor behavior; however this isomer as well as the I_h_ is expected to be very unstable. This isomer also has a very near energy value between HOMO and LUMO, therefore the triplet state was calculated as well as two more cases indicated above, again the excited state is found and the energy difference between it and the ground state is only 2.82 eV. 

The molecular orbital scheme suggests that the most likely stable species are those conformations that have been reported as prepared species. Thus the important question must refer to assessing the stability of I_h_ and likewise the other unstable species must be compared to the pristine prepared isomers, in order to define the expedience of energy comparisons using homodesmotic reactions for this analysis. These reactions have been designed following the same criteria of George and his coworkers [28,29] who proposed for the first time this kind of process. In the present case the task is divided in two parts, in the first one the relative energies of the pristine cages have been calculated from the enthalpy changes using the homodesmotic reaction. In the second part a similar procedure is carried out for the estimation of the enthalpy changes corresponding to the endohedral complexes by means the isodesmic reaction. 

**Scheme 1:**
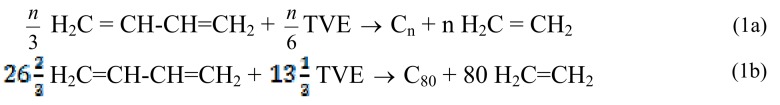
Homodesmotic reaction.

**Scheme 2:**
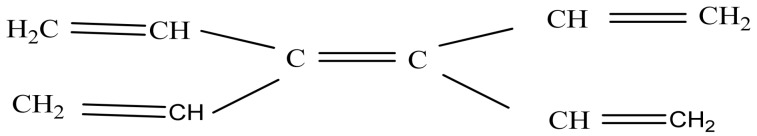
TVE.

The homodesmotic reaction has been described previously [30,31], but was only applied to C_60_ and C_70_ fullerenes, whereas in this work it is also applied to all C_80_ isomers. The general form of the reaction is shown in Scheme 1a; whereas relating to this specific case it is presented in Scheme 1b. The formula of TVE is shown in Scheme 2.

This reaction was chosen because the results previously obtained [30] were compared with other family of results of our group which were validated with other studies, mainly those of Liebman and his coworkers [32,33]. The results for all conformations are presented in Table 1 as well as the relative difference when compared to the more stable isomer (D_5d_).

Firstly, the values are so similar when compared to those in the semi-empirical study [13], that it is possible to conceive of every isomer being attainable; even those from I_h_ and D_5h_. Furthermore, the C_2v_ isomer has been detected as an ion, taking into account the close vicinity of its molecular orbitals. Likewise it requires the application of a very small quantity of energy to form a stable species, indicating that this species is attainable. However this situation indicates a dramatic change when the lanthanum ions are placed into the spheres, as analyzed in the next section.

### 2.2. Endohedral Lanthanum Complexes

**Figure 6:**
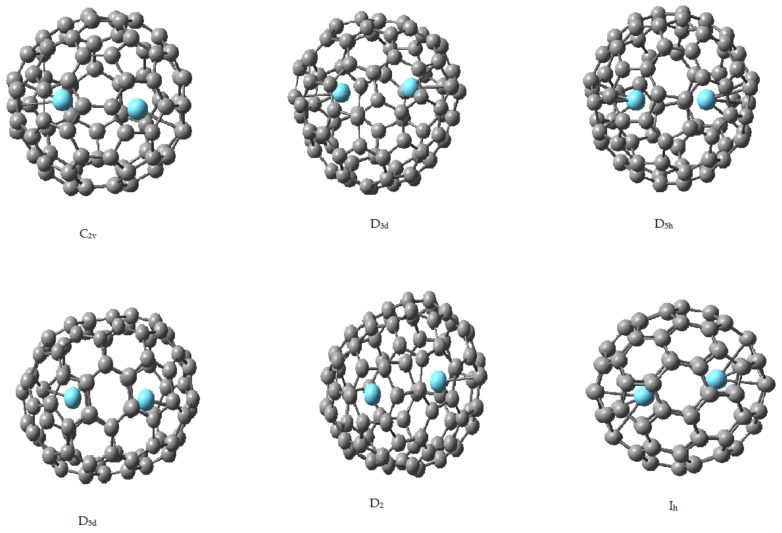
The six isomers of the endohedral complex of Lanthanum and C_80_.

The six endohedral complexes coming from the interaction of two lanthanum ions and each one of the isomer cages of C_80_ are shown in Figure 6.

The evaluation of the stability of the endohedral complexes is also based on a reaction, but in this case it is isodesmic; a process which is depicted in the next scheme, first the general equation:

(2a) $$2{\text{C}}_{n}/\frac{3}{13}\cdot {\text{H}}_{n}/\frac{3}{13}L{\text{a}}_{2}+n/2\frac{2}{3}{\text{C}}_{10}{\text{H}}_{12}\to 2{\text{C}}_{n}{\text{La}}_{2}+n/2\frac{1}{3}{\text{C}}_{2}{\text{H}}_{4}$$

And next the particular case used in this work:

2C_26_H_26_La_2_ + 33.5C_10_H_12_ → 2C_80_La_2_ + 113.5C_2_H_4_ (2b)

This process describes the reaction between tetravinylethylene (TVE) and an organometallic compound containing two lanthanum ions that yields our endohedral complexes and ethylene. This selection of molecules needs to be explained.

Firstly tetravinylethylene is a source of secondary carbon atoms that do not contain hydrogen [30,31]. Its formula is presented in the next scheme where it is possible to observe the two central atoms of carbon that have a double bond between them, even though they are joined to four vinyl groups. Thus these central atoms present a similar pattern to that found on the lattice of a fullerene molecule. 

**Figure 7:**
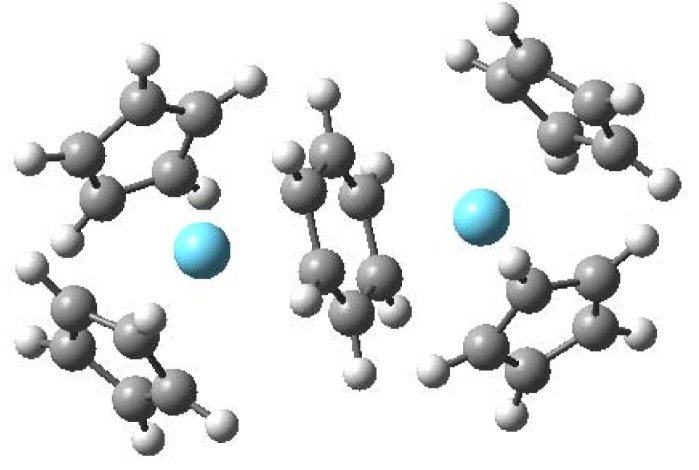
Organometallic lanthanum complex.

The second choice comprises a system that has been mentioned to consist of an anion in the synthesis of organometallic adducts [34], whose shape is presented in Figure 7. This molecule is a source of lanthanum within an organometallic environment and can be used as a generator of π bonds between lanthanum and carbon atoms. It is important to note that the species employed in the present work is neutral because the experimental one has only been reported as an anion, besides in the present case the molecule has no substituents, implying that the central phenyl ring and the four cyclopentadienyl anions contain only hydrogen atoms at the periphery, the general equation can be used for homodesmotic reactions of other endohedral organometallic lanthanum complexes, this will be the theme of a future investigation of our group.

There are two important features to consider. Firstly, the reaction is isodesmic because the lanthanum organometallic compound of the reactive zone contains La(II) ions and the lanthanum cations within the fullerene cages are La(III). This difference is sufficiently marked to suggest that the proposed reaction is isodesmic. Secondly, it has been suggested that the lanthanum ions within the cage display movement in the stabler cages of C_80_ [35,36]. However in the present instance the calculation results have indicated that they tend to join themselves to the inner wall of carbon, a characteristic which needs to be emphasized because obviously this formation of bonds accounts for the absolute value of the estimated energy. There are small differences between the bonds which we will analyze later. The relative energy values are presented in Table 2.

The differences are very interesting as firstly the values shown in Table 1 are all negative; therefore it is possible to conclude that these cages derive additional stability from the energy emanating from either a better or worse electronic distribution. This additional stability has been attributed to a type of spherical aromaticity in C_60_ [37], from which we can conclude that a similar effect occurs in C_80_, in spite of the symmetry or changes in symmetry that are evident in the different cages. 

In a completely different scheme, the organometallic derivatives display positive values and a very different order, the conclusion from these results is that the extra stabilization energy is lost because there are now two positive ions within the cage and the cage itself is an anion, in fact the species are zwitterionic, so the discussion in this case is focused in a different feature that deals with the nature of the interaction among the lanthanum ions and the cage.

First, a big difference between the case of the pristine fullerenes and the endohedral complexes is the order, now the more stable species is the I_h_, in marked contrast to the empty cages. The values shown in Table 2 indicate that the tendency to distortion found in the empty spheres is negated, and that the isomers coming from the cages with pronounced symmetry are the more stable ones, a phenomenon that has been observed previously [38]. The elusive structures of the empty cages are feasible when the lanthanide ions are included within the spheres.

**Figure 8:**
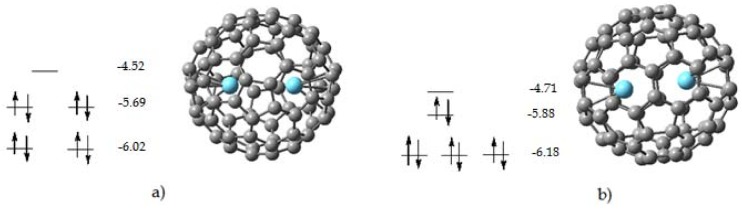
Molecular orbital diagrams for the most symmetric isomers of the endohedral complex (**a**). I_h_; (**b**). D_5h_.

Some examples of molecular orbital diagrams of the endohedral complexes are presented in Figure 8, the arrangement in the case of the I_h_ isomer has been little adapted because the actual situation is those suggested by Nagase and his coworkers [26] in which the point group after the lanthanum doped is D_2h_ and HOMO set is formed by four non-degenerated single MO’s with ag, b_1g_, b_2g_ and b_3g_, however the energy differences among the four orbitals suggest again a kind of accidental degeneracy in two sets, the triple degenerated set of the D_5h_ species come from a similar situation because the definitive point group after the lanthanum doped is C_2_ and this point group obviously has not triple degenerates sets.

The comparison between these and those from the empty cages is important, because the pronounced symmetry and the concomitant accidental degeneracy obtained for the empty species is not the same as that marked in the case of the endohedral complexes. The nature of the HOMO’s is similar to the cages considering the significant contribution made by the cages in all cases, however the important point is that the lanthanide atoms do participate and it is even possible to find strong interactions between these atoms and the inner wall of the cages. Furthermore in almost all cases, the space between lanthanum and the nearest neighboring carbon atom is found to measure between 2.5 and 2.6 Å; values which fall within the interval of the normal bond between La and C [39]. Thus it would seem that lanthanum ions are fixed to the inner wall on the basis of our calculation, this situation has been reported early in experimental as well as theoretical work [40,41] and some interesting recent works have mentioned some similar behavior for several endohedral complexes [42,43], indeed the original idea of strong repulsion between both lanthanum positive ions [44] has changed because it has been demonstrated [41] that the transfer of electrons from lanthanum to the cage can be partial, therefore it is possible to have some amount of covalent bond between the lanthanum atoms. The indications of motion among the lanthanum particles within the cages cited above were considered in studies where Sc or Ce are the species involved. In the present case lanthanum represents the largest ion of the family with apparently limited movement and a tendency to bond to the inner surface.

Both features were considered in the present study, the interaction between lanthanum ions and the cage and the interaction between both lanthanum atoms. The results are shown in Table 3, the very interesting point is that the order of the bond length in both cases follows exactly the same tendency of the results obtained by means the isodesmic reaction, the more symmetrical cages I_h_ and D_5h_ have the shortest bond lengths for the bond of lanthanum with the cage as well as the bond between lanthanum ions, particularly the results of the I_h_ case are very interesting because they are La-C 2.47 Å and La-La 3.71 Å respectively, the first value is shorter than the x-ray result [39] which is ~2.63 Å and the second is shorter than the one previously calculated (3.826 Å).

**Figure 9:**
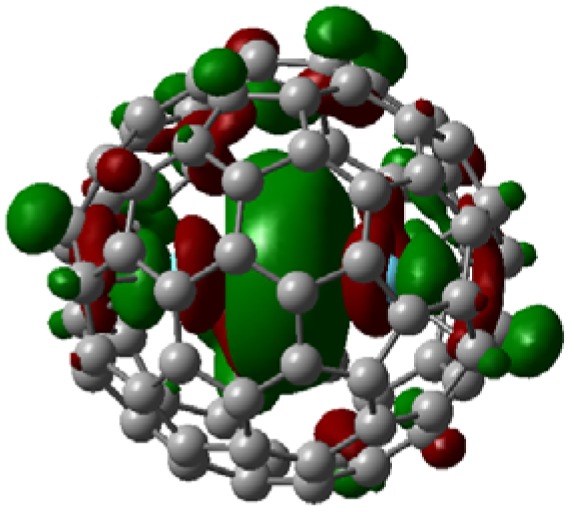
LUMO of D_5h_ Lanthanum complex.

The point is that the extra stabilization factor that changes completely the order in the series of the endohedral complexes is the formation of bonds, the stronger the bond the stabler species. It is important to point out that the LUMO of almost all the endohedral complexes is similar and that it is possible to observe a strong anti-bonding interaction at the center of the cages and in the middle of the distance between both lanthanum particles (see Figure 9), Popov and his coworkers have shown a similar interaction [41]. This is a very interesting observation for two reasons. Firstly it appears that this function arises owing to the repulsion between both ions forcing them to get closer to the inner wall, but secondly there is a very strong anti-bonding lower orbital in all cases, which should thus act as a bridge inducing a kind of back-bond effect.

The empty cages and the endohedral complexes manifest more differences than similarities, and both families definitely comprise very interesting kinds of species. Both cases are the subject of very important studies and offer possibilities for important future applications.

## 3. Experimental

All structures were optimized in the gas phase using the Gaussian09 package [45]. In this work the M06 density functional [46,47], the 6-311G** basis set for light atoms and in the case of the lanthanum a basis set with TZVP quality SARC [48,49] were used. The frequency calculations were carried out at the same level of theory, in order to confirm that the optimized structures were at the minimum of the potential surface. These frequencies were then used to evaluate the zero-point vibrational energy, the thermal vibrational corrections to the enthalpy and the enthalpy values which were used in the homodesmotic and isodesmic reactions. The first reaction which was previously specifically designed for fullerenes [30,31] was applied for pristine fullerene structures, this is a homodesmotic reaction but it can be classified as a hyperhomodesmotic reaction considering the more recent classification [50], however it is still named only homodesmotic, the second one should be classified as isodesmic because it involves lanthanum atoms and shows a change in the oxidation states in both sides of the reaction.

## 4. Conclusions

A computational study focusing on the energetic differences of six isomers of C_80_ as well as their lanthanum complexes has been achieved. The analysis of the molecular orbital arrangements implies that the parent isomer is likely to represent an isomer manifesting I_h_ symmetry. However this appears to have undergone some kind of Jahn-Teller distortion breaking its symmetry and stabilizing the isomer belonging to the D_5d_ and D_2_ point groups. The relative energy for all these species has been computed using a homodesmotic reaction, and the results indicate once again that D_5d_ and D_2_ are the preferred isomers. The isomer D_5h_ comprises a totally distinct conformation from that of the rest of the family. The endohedral complexes manifest very different behavior; the Ih and D5h isomers are unstable when they are empty cages, however they represent the best possibilities as lanthanum complexes, besides almost all of them present strong interaction between the lanthanum particles and the inner wall suggesting a bond. The LUMO in all the complexes displays the same shape, wherein a strong anti-bonding interaction between the two lanthanum particles is apparent. This orbital can work as a bridge for back-bonding.

## Figures and Tables

**Table 1:** Absolute enthalpies and relative energies of the isomers of C_80_ (kcal/mol).

Isomer	∆H	Rel. energy
D_2_	−153.75	0.0
D_5d_	−152.37	3.83
C_2v_	−146.23	5.94
D_3_	−145.26	8.76
D_5h_	−144.34	11.25
I_h_	−128.03	20.98

**Table 2:** Absolute enthalpies and relative energies of the isomers of La_2_@C_80_ (kcal/mol).

Isomer	∆H (kcal/mol)	Rel. energy
I_h_	80.82	0.0
D_5h_	118.80	11.55
C_2v_	155.26	19.54
D_3_	188.40	63.27
D_2_	209.75	88.87
D_5d_	227.21	97.89

**Table 3:** Bond lengths in the isomers of La_2_@C_80_ (Å).

Isomer	C-La	La-La
I_h_	2.470	3.710
D_5h_	2.527	3.735
C_2v_	2.550	3.748
D_3_	2.561	3.928
D_2_	2.570	4.005
D_5d_	2.584	4.150
